# Recurrent urinary tract infection is not one disease: moving from antibiotic suppression to phenotype-guided prevention

**DOI:** 10.1097/MS9.0000000000005446

**Published:** 2026-08-05

**Authors:** Bilal Ahmad, Kaleem Khan

**Affiliations:** a Core Surgical Training, CST-2, Department of Surgery, York & Scarborough Teaching Hospitals NHS Foundation Trust, York, UK; b Department of Surgery and Urology, Balkh University, Mazar-i-Sharif, Balkh, Afghanistan

**Keywords:** antibiotic prophylaxis, antimicrobial stewardship, methenamine hippurate, recurrent urinary tract infection, vaginal estrogen

## Abstract

Recurrent urinary tract infection (UTI) is commonly defined by episode frequency, but recurrence count alone does not explain why infections recur or which preventive strategy is most appropriate. In everyday practice, patients with postmenopausal hypoestrogenism, post-coital recurrence, low fluid intake, voiding dysfunction, persistent-source relapse, resistant-organism recurrence, or noninfectious urinary symptoms may all receive the same label of recurrent UTI despite having different clinical drivers. This editorial argues that recurrent UTI should be approached as a heterogeneous, phenotype-driven syndrome rather than as a uniform pathway toward repeated antimicrobial treatment or long-term antibiotic suppression. Antibiotics remain essential for acute bacterial infection and selected prophylactic use, but prevention should be preceded by confirmation of recurrent bacterial infection and assessment of the dominant recurrence phenotype. Evidence supporting vaginal estrogen, methenamine hippurate, increased fluid intake in selected patients, and cranberry products illustrates the potential value of targeted non-antibiotic prevention, while negative trial evidence for d-mannose reinforces the need for evidence discipline rather than indiscriminate supplement use. We propose a practical model of recurrent UTI care based on four steps: confirm, phenotype, prevent, and reassess. This approach aligns clinical management with antimicrobial stewardship, improves diagnostic clarity, and encourages prevention strategies matched to individual patient mechanisms rather than recurrence count alone.

## Introduction

Recurrent urinary tract infection (UTI) is usually counted before it is understood. Standard definitions are clinically useful because they identify patients with repeated symptomatic episodes and provide a threshold for further evaluation, prevention, and follow-up. However, the recurrence count alone does not explain why infections recur, nor does it identify the most appropriate preventive strategy[1]. A postmenopausal woman with genitourinary syndrome of menopause, a young woman with post-coital episodes, an older man with incomplete bladder emptying, and a patient with persistent urinary symptoms but sterile cultures may all be described as having recurrent UTI. Yet these presentations differ substantially in pathophysiology, diagnostic uncertainty, and management priorities. Treating them as a single entity risks narrowing prevention to repeated antimicrobial exposure while overlooking modifiable drivers of recurrence[2].

This distinction is increasingly important as recurrent UTI care moves beyond simple suppression toward diagnostic accuracy, antimicrobial stewardship, and patient-centered prevention. The American Urological Association (AUA) guideline frames recurrent UTI management around reducing inappropriate antibiotic use, antibiotic resistance, antibiotic-related adverse effects, and the quality-of-life burden[2]. Similarly, the National Institute for Health and Care Excellence (NICE) guideline on recurrent UTI antimicrobial prescribing emphasizes optimization of antibiotic use and reduction of antimicrobial resistance[3]. These priorities do not diminish the role of antibiotics, which remain necessary for acute infection and appropriate for selected prophylactic strategies. Rather, they highlight the need for a more structured clinical approach before long-term suppression is chosen. This editorial argues that recurrent UTI should be approached as a heterogeneous, phenotype-driven syndrome in which the central clinical question shifts from “Which prophylactic antibiotic should be used?” to “What is driving recurrence in this patient?” Episode count should identify recurrence, but phenotype should guide prevention.

## Recurrent UTI is a definition, not a complete diagnosis

Recurrent UTI is best understood as a clinically useful threshold rather than a complete diagnostic explanation[4]. A frequency-based definition helps identify patients who need closer assessment, preventive counseling, and longitudinal follow-up, but it does not establish why recurrence is occurring. The same label may apply to a postmenopausal woman with estrogen deficiency, a patient whose episodes follow sexual activity, an individual with low fluid intake, an older patient with incomplete bladder emptying, a person with a persistent nidus, such as a stone or foreign body, or a patient with urinary symptoms but repeatedly negative cultures. These groups may share recurrence, but they do not share the same mechanism or prevention pathway. Moving directly from recurrence count to long-term prophylaxis therefore risks compressing distinct clinical problems into a single therapeutic response[5]. The diagnosis of recurrence answers “how often?” Phenotyping answers the more clinically useful question: “Why this patient, and why now?”

## Antibiotic suppression works, but it should not be the default endpoint

Antibiotic prophylaxis remains an important option in recurrent UTI care and should not be dismissed. For selected patients with culture-confirmed, frequent, and burdensome infections, continuous or post-coital prophylaxis can reduce recurrence and improve quality of life. The concern is not that antibiotic suppression is ineffective, but that it may be reached before the recurrence phenotype has been adequately defined. This distinction has become more important as non-antibiotic preventive strategies have gained stronger clinical relevance. In the alternatives to prophylactic antibiotics for the treatment of recurrent urinary tract infections in women (ALTAR) randomized non-inferiority trial, methenamine hippurate showed preventive efficacy comparable to daily low-dose antibiotic prophylaxis in women with recurrent UTI, supporting its role as an antibiotic-sparing option in appropriately selected patients[6]. The NICE now recommends considering methenamine hippurate as an alternative to daily antibiotic prophylaxis for recurrent UTI in women and people with a female urinary system, while advising specialist input in pregnancy, recurrent upper UTI, complicated lower UTI, and male genitourinary anatomy[7]. These developments do not replace antibiotics; rather, they reinforce the need to use them deliberately, after confirming the infection pattern, recurrence drivers, patient risk, and the availability of reasonable alternatives.

## The phenotypes that should change management

A phenotype-guided approach begins by asking which clinical pattern should change the prevention plan. In peri- and postmenopausal women, recurrent cystitis may reflect hypoestrogenic changes in the lower genitourinary tract. In this setting, repeated courses of antibiotics alone may treat individual episodes while leaving a modifiable susceptibility factor unaddressed. This is why guideline recommendations for vaginal estrogen are clinically important: they shift prevention from antimicrobial suppression toward restoration of a local biological defense when there is no contraindication.

A second common pattern is post-coital recurrence, in which episodes are temporally related to sexual activity. Recognizing this phenotype can prevent escalation to continuous prophylaxis when a more targeted strategy may be sufficient. Similarly, in premenopausal women with low baseline fluid intake, increased hydration may be a simple antimicrobial-sparing intervention. It should not be presented as a universal solution, but it is clinically meaningful because it matches a low-risk behavioral intervention to a plausible and identifiable driver[8].

Other phenotypes require clinicians to look beyond uncomplicated cystitis. Rapid recurrence with the same organism should raise concern for relapse or a persistent source, including stones, foreign material, obstruction, or incomplete eradication. In these patients, repeated short antibiotic courses may temporarily suppress symptoms while delaying source recognition. Voiding dysfunction represents another important phenotype, particularly in older men, patients with neurogenic bladder, pelvic organ prolapse, or elevated post-void residual volume^[^6,7^]^. The preventive priority is not simply another antimicrobial course but correction or mitigation of impaired emptying.

A resistant-organism phenotype is increasingly relevant in patients exposed to multiple empirical or prophylactic antibiotics. Here, culture-guided treatment, avoidance of unnecessary broad-spectrum agents, and consideration of appropriate non-antibiotic options become central to stewardship. Finally, the mimic phenotype must not be overlooked. Patients with dysuria, urgency, pelvic discomfort, or frequency but with repeatedly negative cultures may have bladder pain syndrome, overactive bladder, vaginitis, urethral syndrome, or pelvic floor dysfunction[9]. In such cases, continued antibiotic cycling may delay the correct diagnosis and add avoidable harm. The educational value of phenotyping is therefore practical: it links the label of recurrent UTI to a management decision that would otherwise be missed.

## Non-antibiotic prevention should be matched to phenotype, not used randomly

Non-antibiotic prevention should not be treated as a single interchangeable category. Its value depends on patient selection, biological plausibility, and trial evidence. Methenamine hippurate is most persuasive when used as an antibiotic-sparing strategy in appropriately selected patients, particularly after recurrent bacterial infection has been confirmed and complicated features have been considered. The ALTAR trial supports this role by showing comparable preventive efficacy to that of daily low-dose antibiotic prophylaxis in women with recurrent UTIs[6]. Cranberry products provide another example of selective use. The 2023 Cochrane review, which included 50 randomized trials and 8857 participants, found a reduction in UTIs among women with recurrent UTIs, children, and people susceptible to UTIs after interventions, while not supporting routine use in some groups, such as elderly patients, pregnant women, or those with bladder emptying problems[10].

D-mannose illustrates the opposite lesson and strengthens the argument for evidence discipline. Although widely used, a 2024 randomized clinical trial in primary care found that daily d-mannose did not reduce medically attended UTI among women with recurrent UTI and should not be recommended for prophylaxis in that setting[11]. A phenotype-guided approach therefore does not simply replace antibiotics with supplements. It matches prevention to mechanism, uses non-antibiotic options where the evidence and patient profile support them, and rejects interventions when trial evidence is weak or negative.

## A practical model: confirm, phenotype, prevent, reassess

A phenotype-guided approach can be translated into a simple clinical sequence: confirm, phenotype, prevent, and reassess[12]. First, clinicians should confirm that the patient truly has recurrent bacterial UTI by documenting symptoms, urine culture results, organism pattern, and resistance profile wherever feasible. This step is essential because recurrent urinary symptoms are not always due to recurrent infection. Second, the recurrence phenotype should be defined by asking why this patient is experiencing recurrences: estrogen deficiency, post-coital triggering, low fluid intake, voiding dysfunction, relapse from a persistent source, resistant-organism recurrence, or a noninfectious mimic. Third, prevention should be matched to the dominant phenotype rather than chosen generically. This may include vaginal estrogen, hydration, methenamine hippurate, cranberry products in selected patients, post-coital strategies, correction of impaired bladder emptying, source control, or selective antibiotic prophylaxis. Finally, the plan should be reassessed based on recurrence frequency, culture pattern, adverse effects, development of resistance, quality-of-life burden, and need for specialist referral. The prevention plan should be reviewed like any other long-term therapy: with a defined indication, measurable outcome, adverse-effect assessment, and exit strategy.

## Conclusion

Recurrent UTI should not be reduced to repeated antibiotic treatment or automatic progression toward long-term suppression. Antibiotics remain essential for acute bacterial infection, and prophylaxis remains valuable for selected patients with confirmed, frequent, and burdensome recurrence. However, prevention is most clinically meaningful when preceded by confirmation and phenotyping. This approach helps distinguish patients who may benefit from local estrogen therapy, behavioral modification, antibiotic-sparing prophylaxis, culture-guided antimicrobial strategies, urologic evaluation, or reassessment for noninfectious mimics. It also aligns recurrent UTI care with antimicrobial stewardship without denying the legitimate role of antibiotics. Recurrent UTI should no longer be approached as a countdown to prophylaxis but as a signal to identify the phenotype driving recurrence and prevent the next infection more intelligently.

## Ethical approval

Not applicable. This article is an editorial based exclusively on previously published literature and did not involve human participants, animals, or identifiable patient data.

## Consent

Not applicable. No patients or identifiable personal information are included in this article.

## Sources of funding

The authors received no financial support or funding for the research, authorship, or publication of this article.

## Data Availability

Data sharing is not applicable to this article, as no datasets were generated or analyzed during the preparation of this editorial.

## Research registration unique identifying number

Not applicable. This article is an editorial and does not involve a clinical trial, systematic review registration, or prospective research registration requiring a unique identifying number.
